# UNEXPECTED CAREGIVING: LIFE AFTER A COVID-19 INTENSIVE CARE UNIT HOSPITALIZATION

**DOI:** 10.1093/geroni/igac059.474

**Published:** 2022-12-20

**Authors:** Sheria Robinson-Lane, Florence Johnson, Amanda Leggett, Alicia Carmichael, Natalie Leonard, Richard Gonzalez

**Affiliations:** University of Michigan School of Nursing, Ann Arbor, Michigan, United States; University of Michigan, Ann Arbor, Michigan, United States; Wayne State University, Ypsilanti, Michigan, United States; University of Michigan Institute for Social Research, Ann Arbor, Michigan, United States; University of Michigan Institute for Social Research, Ann Arbor, Michigan, United States; University of Michigan, Ann Arbor, Michigan, United States

## Abstract

The coronavirus pandemic has led to an exceptional number of critical care hospitalizations followed by extended recovery periods that necessitate familial support. Using a qualitative descriptive approach, this study aimed to examine the strategies used by families to adjust to the caregiving role. Semi-structured interviews of patients who had been recently discharged from the Intensive Care Unit (ICU) (n=16) along with their family caregivers (n=16) were thematically analyzed. Three major themes were identified that highlight how family caregivers adapt to the caregiving role following an ICU COVID-19 related hospitalization including 1) engaging the support of family and friends, 2) shifting responsibilities to accommodate caregiving, and 3) managing one’s emotions. Additional themes more specifically related to managing COVID-19 care included: 1) managing infection control, 2) care recipient’s need for independence, and 3) managing support services. Flexibility and sufficient support allowed family caregivers to manage their new caregiving role and function optimally.

